# Is There a Risk of Lymphoma Associated With Anti-tumor Necrosis Factor Drugs in Patients With Inflammatory Bowel Disease? A Systematic Review of Observational Studies

**DOI:** 10.3389/fphar.2019.00247

**Published:** 2019-03-19

**Authors:** Sara Ferraro, Luca Leonardi, Irma Convertino, Corrado Blandizzi, Marco Tuccori

**Affiliations:** ^1^Unit of Pharmacology and Pharmacovigilance, Department of Clinical and Experimental Medicine, University of Pisa, Pisa, Italy; ^2^Unit of Adverse Drug Reaction Monitoring, University Hospital of Pisa, Pisa, Italy

**Keywords:** anti-TNF, lymphoma, observational study, inflammatory bowel disease, Crohn's disease, ulcerative colitis

## Abstract

**Background:** Inflammatory bowel diseases (IBDs) are generally not considered a risk factor for the development of lymphoma. When considering IBD treatments, there is good evidence supporting thiopurines (azathioprine, 6-mercaptopurine) as a risk factor for lymphoma. Conversely, the association between the use of anti-TNF agents and the development of lymphoma remains undetermined. In this systematic review, we analyzed the evidence coming from observational studies supporting an association between the use of anti-TNF drugs and lymphoma in patients with IBDs.

**Methods:** This systematic review was performed according with MOOSE and PRISMA statements. We searched observational studies conducted on IBD patients, using MEDLINE, EMBASE, and Google Scholar, published in English language, within the period ranging from January 1st, 1999 to June 30th, 2018. An assessment of the methodologic shortcomings of selected studies was performed as well.

**Results:** Fourteen studies met the eligibility criteria and were included in the review. Only four studies found a significant association of anti-TNF drug with lymphoma or groups of cancers including lymphoma. However, the methodologic shortcomings of all the included studies made their results unreliable, irrespectively of whether their findings supported an association or not.

**Conclusions:** Current evidence from observational studies does not allow excluding or confirming an association of the exposure to anti-TNF treatments with lymphoma in IBD patients.

## Introduction

The term “inflammatory bowel disease” (IBD) describes a group of immune disorders characterized by chronic inflammation of the digestive tract. The main types of IBD include ulcerative colitis (UC) and Crohn's disease (CD) (Khor et al., 2011). The major complications associated with IBDs are thrombosis, primary sclerosing cholangitis, skin, eye and joint inflammation and even colonic cancer (Rothfuss et al., 2006). When considering other neoplastic complications, there is some evidence that chronic inflammation might be a risk factor for lymphoma (Ekström Smedby et al., 2008). However, at variance with other immune-mediated inflammatory disorders, such as rheumatoid arthritis (RA) (Simon et al., 2015; Mercer et al., 2017), the evidence supporting an association of IBDs with the development of lymphoma is still scarce (Williams et al., 2014). Furthermore, some authors suggested that pharmacological treatments for IBDs and RA (e.g., anti-tumor necrosis factor drugs, TNF), could promote the development of lymphoma (Herrinton et al., 2011; Parakkal et al., 2011). However, owing to the intrinsic risk for background diseases, it is difficult to establish an association between pharmacological treatments and the onset of lymphoma in these categories of patients, as well as to identify clear underlying determinants of biological plausibility (Baecklund et al., 2014).

When IBDs treatments have been considered in details, thiopurines (azathioprine, 6-mercaptopurine) were found to increase the risk of lymphoma (Kotlyar et al., 2015), while the association between the use of anti-TNF agents and the development of lymphoma remains questionable (Herrinton et al., 2011; Lichtenstein et al., 2012; Nyboe Andersen et al., 2014; Williams et al., 2014). A disproportionality analysis conducted on suspected adverse drug reactions recorded in the FDA Adverse Event Reporting System (FAERS) on patients with IBD, suggested a signal of risk for thiopurines, alone or in combination with anti-TNF drugs, but not with anti-TNF drugs alone (Deepak et al., 2013). Moreover, data from randomized clinical trials (RCT) are generally conflicting, likely because of the long term and rarity of the outcome (Chen et al., 2016). Likewise, available observational studies have provided conflicting results. In this regard, a recently published observational study (Lemaitre et al., 2017) showed a significant risk of lymphoma in patients with IBDs receiving anti-TNF monotherapy, thiopurine monotherapy or combination therapies, as compared with unexposed patients, thus fostering further the debate about the safety surrounding these treatments.

In light of the above-mentioned conflicting knowledge, we performed a systematic review of observational studies in patients with IBDs, focused on the association of lymphoma with the use of anti-TNF drugs, whatever the comparator, in order to analyze the solidity of evidence supporting this relationship.

## Methods

### Search Strategy and Study Selection

The present systematic review was performed in accordance with PRISMA (Shamseer et al., 2015) and MOOSE (Stroup et al., 2000) statements. We conducted a literature search in MEDLINE, EMBASE, and Google Scholar by a combination of the following keywords: (“infliximab” OR “adalimumab” OR “certolizumab pegol” OR “golimumab”) AND (“lymphoma”). We examined databases for all indexed articles, restricted to the English language, with publication dates falling in the period from January 1st, 1999 (year of infliximab approval) to June 30th, 2018. Duplicates were removed primarily by Mendeley auto-deduplication tool and then by manual assessment. Three reviewers (I.C., S.F., L.L.) examined the retrieved papers. The reviewers assessed the relevance of the collected studies by the title and abstract. If the study eligibility remained unclear, the full text was checked. Any disagreement was resolved by discussion with a senior reviewer (M.T.).

### Study Inclusion and Exclusion Criteria

We included only observational studies that evaluated the risk of lymphoma associated with the four TNF inhibitors of interest, namely infliximab, adalimumab, certolizumab pegol and golimumab, currently approved for treatment of patients affected by CD and UC. In particular, studies were included only if they reported lymphoma incident rate ratio (95% Confidence Interval [CI]) or hazard ratio (95% CI) in CD or UC patients exposed to anti-TNF drugs. We did not consider any restriction about comparator groups. Notably the accepted studies reported lymphoma as specific (all types of lymphoma) or composite (i.e., lymphoma was included in a larger cluster of malignancy) outcomes. However, we included studies with composite endpoints only when they reported the overall number of lymphoma cases. Articles focused exclusively on special populations (i.e., pediatric patients) were excluded. Review articles, randomized trials, open-label extension studies, case series, articles based on questionnaires, case reports, unpublished studies, and conference abstracts were not included.

### Study Classification and Definitions

The following information were extracted from each selected study: authors and publication year, type of source used to collect patient clinical data, design and main methodologic characteristics (information about adjustments and matchings, presence of lag period, inclusion of prevalent patients -considered as patients who were already users of the drug of interest at the cohort entry-), observation period, patient disease (IBD, CD or UC), and drug exposure. The observation period was defined by the time interval in which patients were followed for the outcomes of interest. The lag period was defined as the time window in which a patient should be considered “not exposed” to the potential risk factor (i.e., a drug) for the event of interest, since the temporal relationship would not be supportive of a causative role. Each study was read in full by two experts and the study designs methodology was assessed carefully by expert judgment, particularly for relevant biases, such as immortal time bias (Targownik and Suissa, 2015) and time-window bias (Suissa et al., 2011). Any disagreement was resolved with a third expert. We extracted also outcome measures [number of lymphoma events, number of person-year, incidence rate (95% CI) and hazard ratio (95% CI) values], when available. This review was not submitted in advance to any public repository.

## Results

**Figure 1:**
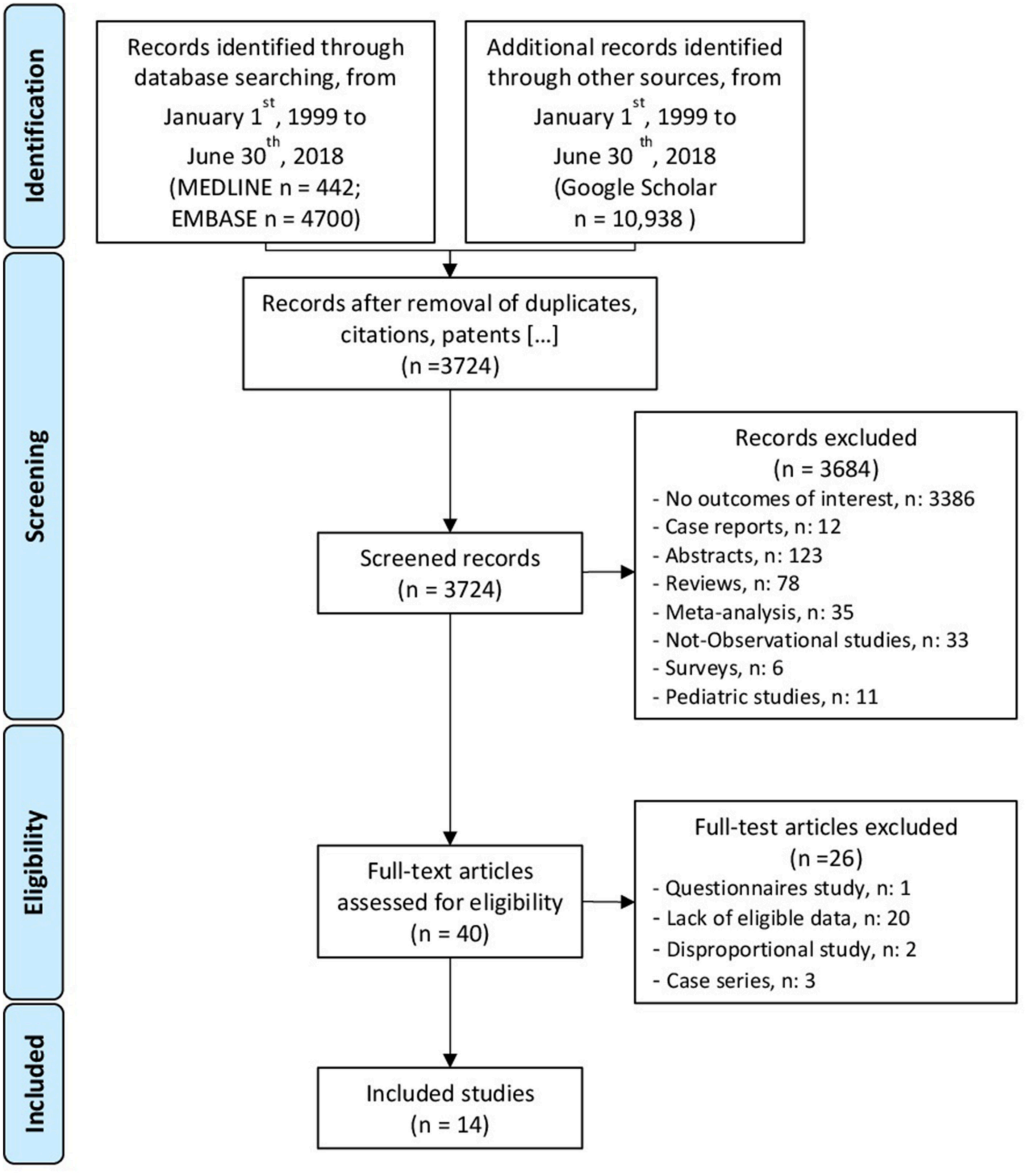
PRISMA (Shamseer et al., 2015) flow chart of the search result of the analysis.

Figure 1 summarizes the search strategy and the selection process. Among 3,724 screened records, 3,684 were excluded after reviewing title and abstract. Forty-one full-text publications were assessed for eligibility. Overall, fourteen full-text articles met the eligibility criteria and were analyzed in detail. Table 1 summarizes the characteristics of the included studies. Among the selected articles, five included lymphoma as a specific endpoint. Nine studies (Biancone et al., 2006, 2016; Fidder et al., 2009; Haynes et al., 2013; Beigel et al., 2014; Lichtenstein et al., 2014; Nyboe Andersen et al., 2014; Liu et al., 2015; D'Haens et al., 2017) performed an assessment of cancer risk that included lymphoma cases among the endpoints, but only two (Lichtenstein et al., 2014; Liu et al., 2015) of them provided a specific assessment of lymphoma (Tables 2A,B).

**Table 1:** Characteristics of the selected studies.

**References**	**Design**	**Data source**	**Observation Period**	**Patients (*n*, disease)**
Biancone et al., 2006	Prospective matched pair cohort study	Italian IBD referral centers	1999–2004	○ 404, CD patients exposed to infliximab ○ 404, CD patients never exposed to infliximab
Fidder et al., 2009	Retrospective cohort study	Medical records of the Gasthuisberg University Hospital, Belgium	1994–2008	○ 743, IBD patients exposed to infliximab ○ 666, IBD patients unexposed to infliximab
Herrinton et al., 2011	Retrospective cohort study	Kaiser Permanente IBD and cancer registry	2000–2006	○ 4,918, CD patients ○ 9,499, UC patients ○ 1,606, IBD not further specified patients
Afif et al., 2013	Nested case-control study	Mayo Clinic Rochester diagnostic index	1980–2009	○ 80, lymphoma cases (44 CD, 36 UC) ○ 159, IBD controls
Haynes et al., 2013	Retrospective cohort study	Medicare and Medicaid databases, Kaiser permanent Northern California Registry	1998–2007	○ 2,657, IBD patients exposed to anti-TNF drugs ○ 3,700, IBD patients unexposed to anti-TNF drugs
Nyboe Andersen et al., 2014	Prospective cohort study	Danish Nationwide Registry	1999–2012	○ 4,553, IBD patients exposed to anti-TNF drugs ○ 51,593, IBD patients unexposed to anti-TNF drugs
Beigel et al., 2014	Retrospective cohort study	Medical records and histopathological reports from a German IBD center	2000–2010	○ 404, IBD exposed to anti-TNF drugs ○ 262, IBD patients never exposed to anti-TNF drugs
Lichtenstein et al., 2014	Prospective cohort study	Crohn' s Therapy, Resource, Evaluation, and Assessment Tool (TREAT) Registry	1999–2010	○ 3,420, CD patients exposed to infliximab ○ 2,509, CD patients unexposed to infliximab
Kopylov et al., 2015	Nested case-control study	Québec health insurance claims database and registry	1996–2009	○ 121 IBD, lymphoma cases ○ 1,201 controls
Liu et al., 2015	Retrospective matched cohort study	Health Core Integrated Research Database, health insurance claims database	2007–2011	○ 515 CD patients exposed to infliximab ○ 515 CD patients exposed to adalimumab and certolizumab pegol
Biancone et al., 2016	Nested case-control study	Clinical records of Italian IBD referral centers	2012–2014	○ 174 IBD malignancy cases (6 lymphoma) ○ 378 IBD controls
D'Haens et al., 2017	Prospective cohort study	ENCORE Registry (European safety registry)	2003–2013	○ 1,541 CD patients exposed to infliximab ○ 1,121 CD patients initially unexposed to infliximab (298 switches to infliximab)
Lemaitre et al., 2017	Retrospective cohort study	SNIIRAM French National Health Insurance claim database	2009–2015	○ 30,294 IBD patients exposed to anti-TNF drugs ○ 50,405 IBD patients unexposed to anti-TNF drugs
D'Haens et al., 2018	Prospective cohort	Multicentre CD registry of adult patients treated with adalimumab: PYRAMID	2007–2015	○ 5,025 CD patients exposed to adalimumab

*IBD, inflammatory bowel disease; CD, crohn's disease; UC, ulcerative colitis*.

**Table 2A:** Studies with lymphoma as specific outcome.

**References**	**Exposure**	**Events (*n*)**	**Person-year (PY)**	**Incident rate (95% CI)**	**Hazard ratio (95%)**
Herrinton et al., 2011	Never anti-TNF and total (never, past, current) thiopurines	38	85.09	44.7 PY	SIR 1.0 (0.96–1.1)
	Past anti-TNF and total (never, past, current) thiopurines	3	2,217	135.3 PY	SIR 5.5 (4.5–6.6)
	Current anti-TNF and total (never, past, current) thiopurines	2	1,757	113.8 PY	SIR 4.4 (3.4–5.4)
Afif et al., 2013	Anti-TNF (infliximab, adalimumab)	9	NA	NA	OR: 2.04 (0.32–12.79)
	Unexposed	71	NA	NA	NA
Lichtenstein et al., 2014	Infliximab	8	17,712	0.05/100 PYs	HR 0.98 (0.34, 2.82) AdjHR: 0.59 (0.28, 1.22)
	Other-treatments-only	6	13,251	0.05/100 PYs	HR 1.00 (reference)
Kopylov et al., 2015	No use of TH/BIO/MTX	92	NA	NA	RR: 1.00
	TP and no BIO/MTX	26	NA	NA	RR: 0.87 (0.53–1.41)
	BIO and no TP/MTX	0	NA	NA	RR: 0
	TP and BIO and no MTX	3	NA	NA	RR: 3.10 (0.72–13.48)
Liu et al., 2015	Infliximab	3	NA	3.3/1,000 PYs	NA
	Adalimumab or certolizumab pegol	1	NA	1.1/1,000 PYs	NA
Lemaitre et al., 2017	Combination Therapy vs. Anti-TNF Monotherapy	14	14,753	0.95/1,000 PYs (0.45–1.45)	AdjHR: 2.35 (1.31–4.22)
	Combination Therapy vs. Thiopurine Monotherapy	14	14,753	0.95/1,000 PYs (0.45–1.45)	AdjHR: 2.53 (1.35–4.77)
	Anti-TNF Monotherapy vs. Unexposed to Thiopurines or Anti-TNF Agents	32	77,229	0.41/1,000 PYs (0.27–0.55)	AdjHR: 2.41 (1.60–3.64)
	Anti-TNF Monotherapy vs. Thiopurine Monotherapy	32	77,229	0.41/1,000 PYs (0.27–0.55)	AdjHR: 0.93 (0.60–1.44)
D'Haens et al., 2018	Adalimumab	10	16.680,4	0,060/100 PYs	NA

*SIR, standardized incident rate ratio; NA, not available; OR, odds ratio; TP, thiopurines; MTX, methotrexate; BIO, biologics; AdjHR, adjusted hazard ratio; RR, relative risk*.

**Table 2B:** Studies with lymphoma included in a composite outcome.

**References**	**Exposure**	**Events (*n*)**	**Person year (PY)**	**Incident rate (95% CI)**	**Risk (95%)**
Biancone et al., 2006	Infliximab	9 (0 lymphoma)	NA	0	NA
	Immunosuppressant not further specified	7 (1 lymphoma)	NA	NA	1
Fidder et al., 2009	Infliximab	23 (2 lymphoma)	3,775	NA	OR: 0.97 (0.56–1.65, *p* = 0.91)
Haynes et al., 2013	Anti-TNF (96.8% of infliximab, 3.2% adalimumab) vs. other immunosuppressant drugs	<5	2,865.3	0.08/100 PYs	HR: 0.41 (0.07–2.35)
Nyboe Andersen et al., 2014	Anti-TNF	8 (6 lymphoma)	18,440	4.34/10,000 PYs	AdjRR: 0.90 (0.42–1.91)
	Not exposed to anti-TNF	260 (NA)	469,874	5.53/10,000 PYs	1
Beigel et al., 2014	TP monotherapy	20 (4 lymphoma)	NA	NA	HR: 4.15 (1.82–9.44)
	TP + Anti-TNF	8 (1 lymphoma)	NA	NA	NA
Biancone et al., 2016	Anti-TNF monotherapy	14 (0 lymphoma)	NA	NA	NA
	Anti-TNF and TP	27 (2 lymphoma)	NA	NA	OR: 2.15 (1.16–4.10) (CD) OR: 0.68 (0.20–2.8) (UC)
	No anti-TNF, No TP	61 (3 lymphoma)	NA	NA	NA
	TP monotherapy	28 (1 lymphoma)	NA	NA	NA
D'Haens et al., 2017	Infliximab vs. conventional therapy	49 (9 lymphoma)	7,362	7.6/1,000 PYs (5.7–9.9)	HR = 1.44; (0.86–2.42, *p* = 0.163)

*TP, thiopurines; OR, odds ratio; HR, hazard ratio; NA, not available; AdjRR, adjusted rate ratio*.

### Lymphoma as Specific Outcome

Among the selected publications, seven studies (Herrinton et al., 2011; Afif et al., 2013; Lichtenstein et al., 2014; Kopylov et al., 2015; Liu et al., 2015; Lemaitre et al., 2017; D'Haens et al., 2018) assessed lymphoma as a specific outcome (Table 2A).

Only two (Herrinton et al., 2011; Lemaitre et al., 2017) out of the seven studies found a significant association of the drugs with the outcomes of interest, and they both examined a general exposure to the class of anti-TNF drugs. Both studies included prevalent patients. Only the study by Lemaitre et al. included a lag period in a sensitivity analysis (Lemaitre et al., 2017). Herrinton et al. estimated the exposure time for both anti-TNF drugs and thiopurines based on treatment coverage while the exposure time after treatment discontinuation was allocated to the unexposed group (Herrinton et al., 2011).

The remaining five studies (Afif et al., 2013; Lichtenstein et al., 2014; Kopylov et al., 2015; Liu et al., 2015; D'Haens et al., 2018) assessing the specific risk of lymphoma in anti-TNF users did not find any association. Three of them evaluated the exposure to the overall anti-TNF class (Afif et al., 2013; Lichtenstein et al., 2014; Kopylov et al., 2015). Two (Afif et al., 2013; Kopylov et al., 2015) out of these three studies included prevalent patients and only one (Kopylov et al., 2015) considered a lag period. The results of all these studies are likely affected by time-related biases. One study (Kopylov et al., 2015) likely had no sufficient power to assess the risk of lymphoma. Only the study by D'Haens et al. (2018) assessed the risk of lymphoma for a specific anti-TNF drug (adalimumab). This study included prevalent patients, did not consider a lag period and assessed the risk of lymphoma in adalimumab patients without a comparison group, but comparing the rate of lymphoma with an estimated background lymphoma rate in the general population, adjusted for thiopurine use. Liu et al. (2015) estimated the frequency of lymphoma in two populations of anti-TNF users, stratified by the route of administration (infliximab and adalimumab/certolizumab pegol, respectively). This study included prevalent patients and was likely not powered enough to detect the risk of lymphoma (Table 3).

**Table 3:** Methodologic features of the elected studies.

**References**	**Adjustment/Matching**	**Prevalent patients (yes, no)**	**Lag period (yes-length, no)**	**Bias assessment**
Biancone et al., 2006	○ Matching ○ Age (± 5 years) ○ Sex ○ Follow up period in the same center (±5 years) ○ Immunosuppressant use (yes/no; type; duration) ○ CD site (ileum, ileum-colon, colon, other) ○ CD duration (±5 years)	Yes	No	Matching is inadequate to control for confounding (i.e., patients receiving infliximab have an average treatment with immunosuppressant drugs of 3 years compared with 2 years in the non-exposed group). The study has not likely the sufficient power to estimate rare endpoints like cancer (specific cancers in particular). It is not clear whether the exposed and not exposed patients are from the same cohort and the possibility of a selection bias is high
Fidder et al., 2009	○ Adjustment ○ Gender ○ Age ○ Weight ○ Disease duration ○ Concomitant immunosuppressive therapy	Yes	No	Possible immortal time bias (patients apparently did not contribute with person time to both exposed and unexposed group).
Herrinton et al., 2011	Not available	Yes	No	Person-time in patients receiving thiopurines and anti-TNF was attributed from the beginning of the treatment to the end of the coverage. The subsequent time was attributed to non-treatment despite the past exposure. This diluted the risk observed in non-exposed patients and concentrated the risk in exposed ones.
Afif et al., 2013	○ Matching ○ IBD patients ○ ± 5 years at the Mayo clinic ○ Subtype of IBD ○ Geographic area ○ Duration of follow-up at Mayo Clinic	Yes	No	Possible time-window bias (follow-up is not from the actual initial IBD diagnosis).
Haynes et al., 2013	○ Matching ○ Propensity score	No	No	–
Nyboe Andersen et al., 2014	○ Adjustment ○ Propensity score matching ○ Year of birth ○ Calendar year of cohort entry ○ Sex ○ Socioeconomic status ○ Degree of urbanization ○ Co-medications (not-IBD)	No	Yes-−3 months (1 year sensitivity analysis)	Cohort entry no clearly stated (likely IBD diagnosis). Not clear whether patients contributed with person time to both exposed and unexposed group. Lag period time was not included in the person-time of unexposed but considered in an unspecified “distinct category” (possible immortal time).
Beigel et al., 2014	○ Adjustment ○ Age ○ Sex	Yes	No	The definition of cohort entry is not clear and we cannot exclude the inclusion of prevalent patients cannot be excluded. The study has not likely the sufficient power to estimate rare endpoints like cancer (specific cancers in particular). Time-fixed analysis with probable immortal time bias.
Lichtenstein et al., 2014	○ Adjustment ○ Age ○ Sex ○ Race	No	No	Immortal time bias: time fixed analysis in which person-time of patients receiving infliximab was classified as exposed to infliximab even before the starting of infliximab treatment
Kopylov et al., 2015	○ Matching ○ Age ○ Sex ○ Duration of disease	Yes	Yes-−1 year (6 months and 2 years in the sensitivity analysis)	Cohort entry definition may expose to the risk of time-window bias. The study has not likely the sufficient power to estimate rare endpoints like cancer (specific cancers in particular).
Liu et al., 2015	○ Adjustment ○ Age ○ Sex	Yes	No	The study has not likely the sufficient power to estimate the risk of lymphoma across groups Despite cohort entry is established at the first drug prescription, we cannot exclude the assumption administration of the drug in over the 6 months preceding the index date (some patients could be prevalent)
Biancone et al., 2016	○ Matching ○ IBD center, ○ IBD type [CD vs. UC] ○ Sex ○ Age [± 5 years]	Yes	No	Possible time-window bias (matching was not performed considering the duration of follow-up)
D'Haens et al., 2017	○ Adjustment ○ Age ○ Disease duration	Yes	No	The definition of cohort entry is not clear and we cannot exclude the inclusion of prevalent patients cannot be excluded.
Lemaitre et al., 2017	○ Adjustment ○ Baseline ○ Time-dependent covariates	Yes	Yes—(3/6 months)	In the sensitivity analysis, the exclusion of person-time associated with lag period did not resolve the problem of the lack of the lag period.
D'Haens et al., 2018	○ Adjustment ○ Exposure	Yes	No	Lack of a comparison group (the authors compared the incidence of lymphoma with an estimated background lymphoma rate in the general population, adjusted for thiopurines use).

*CD, Crohn's disease; IBD, inflammatory bowel disease; UC, ulcerative colitis*.

### Lymphoma as Composite Outcome

Among the selected studies, seven (Biancone et al., 2006, 2016; Fidder et al., 2009; Haynes et al., 2013; Beigel et al., 2014; Nyboe Andersen et al., 2014; D'Haens et al., 2017) reported data on lymphoma outcome included in a broader definition of malignancies (Table 2B).

Two studies (Biancone et al., 2016; D'Haens et al., 2017) provided results supporting an association between the investigated drugs and malignancies including lymphoma. Biancone et al. (2006) investigated the risk of extra-colonic cancer (two lymphoma cases out of 27 events of malignancy) in a population of patients taking any anti-TNF for IBD (Biancone et al., 2016). These authors found a positive association only in the subgroup of CD patients and not in those with UC. This study included prevalent patients, did not consider a lag period and the results are possibly affected by a time-window bias. D'Haens et al. (2017) investigated the risk of lymphoproliferative disorders and malignancies (nine lymphoma cases out of 49 malignancies) associated with infliximab in patients with CD (D'Haens et al., 2017). This study did not consider a lag period and likely included prevalent patients.

Five studies (Biancone et al., 2006; Fidder et al., 2009; Haynes et al., 2013; Beigel et al., 2014; Nyboe Andersen et al., 2014) did not demonstrate an association of anti-TNF drug exposure with the risk of malignancies including lymphoma. Three studies investigated the risk of malignancies in IBD patients exposed to any anti-TNF drug. Andersen et al. assessed the risk of hematopoietic and lymphoid tissue malignancies (six cases of lymphoma out of eight hematological malignancies). The definition of “exposure” might have biased the results due to the inclusion of immortal time (Nyboe Andersen et al., 2014). Beigel et al. investigated the risk of malignancies (one lymphoma out of eight malignancies) in patients receiving both thiopurines and anti-TNF inhibitors. This study included prevalent patients, did not consider a lag period and lacked sufficient power to assess the risk of malignancies. Moreover, results were likely affected by immortal-time bias (Beigel et al., 2014). Haynes et al. assessed the risk of any lymphoma or leukemia in patients exposed to anti-TNF drugs (<5 lymphoma events). In this study the author did not consider a lag period (Haynes et al., 2013).

The remaining two studies investigated the risk of neoplasia (Biancone et al., 2006) (no lymphoma cases in the exposed group and one out of seven in the control group) and of any cancer or dysplasia (two lymphoma cases out of 23 malignancies) (Fidder et al., 2009). Biancone et al. did not observe any risk in CD patients receiving infliximab (Biancone et al., 2006). However, this study included prevalent patients, did not consider a lag period, it could have matching issues and it was likely not powered enough to detect the risk of lymphoma. Fidder et al. investigated IBD patients receiving infliximab (Fidder et al., 2009). In this study, prevalent patients were included, and a lag period was not considered. The results were likely affected by immortal-time bias (Table 3).

## Discussion

Observational studies are usually conducted in an attempt of overcoming the limitations of clinical trials by assessing the long-term effects of medications on infrequent outcomes or in specific sub-populations (Suissa, 2008), such as the risk of lymphoma in IBD patients receiving anti-TNF drugs. The widespread implementation of computer-based health databases, containing routinely collected administrative or clinical data, has encouraged the conduction of observational studies. However, no cautions for managing adequately the methodological underlying such investigations are usually taken. As a consequence, over the last decade, this superficial approach has led to an explosion of the publication of a high number of poorly conceived studies and analytic designs that have generated incorrect or unreliable conclusions on the safety of exposure to drugs (Sherman et al., 2016).

The results of the present systematic review are fully in line with the mentioned above trend. Indeed, very important methodologic issues, such as the inclusion of prevalent patients (11 out of 14 studies) and the lack of an adequate latency period in the definition of exposure (11 out of 14 studies) turned out to be very frequent among the selected studies. The results of seven selected studies were influenced also by important time-related biases, such as time-window bias (Biancone et al., 2006; Afif et al., 2013; Kopylov et al., 2015) and immortal-time bias (Fidder et al., 2009; Beigel et al., 2014; Lichtenstein et al., 2014; Nyboe Andersen et al., 2014). Thus, due to the above limitation, the overall evidence, either supporting the association or not, is strongly conditioned by the methodologic shortcomings of the available studies.

Among the 14 observational studies, only two (Herrinton et al., 2011; Lemaitre et al., 2017) reported data supporting an increased risk of lymphoma in IBD patients treated with anti-TNF, and both have important methodologic shortcomings. Lemaitre et al. estimated a significant relative risk of lymphoma in all treatment groups (thiopurines monotherapy, anti-TNF monotherapy and the combination of thiopurines plus anti-TNF) as compared with unexposed patients (adjusted hazard ratio [aHR]: 2.60; 95% CI, 1.96–3.44; *P* < 0.001; aHR: 2.41; 95% CI, 1.60–3.64; *P* < 0.001; aHR: 6.11; 95% CI, 3.46–10.8; *P* < 0.001, respectively). Of note, the findings of this study are biased at least in part, by the definition of “exposure.” In the main analysis, a lag period was not considered. This means for instance that, if a diagnosis of lymphoma was made few days after the initiation of a treatment with an anti-TNF drug, the adverse event was attributed to the anti-TNF group, despite this outcome is not biologically plausible. In a correct time-dependent analysis, this event would have been attributed to the control group of unexposed patients or to the thiopurine treatment group, depending on whether the treatment with anti-TNF drugs had been a first line or a second line therapy, respectively. With the current analysis, we do not know how many events were attributed to the wrong group of treatment. However, it is likely that the as a ultimate consequence, this bias concentrated most of the event of lymphoma in the treatment groups while diluting the number of these events within the control group, thus leading to an apparent increased risk for all treatments. Of note, in an attempt of controlling for this issue, the authors performed a sensitivity analysis, where they introduced a lag period of 3 and 6 months. Unfortunately, with this approach, they introduced a further bias that apparently confirmed the results of the main analysis. Indeed, instead of attributing the person-time of this lag period to the unexposed group or to the patients exposed to the first-line treatment, they eliminated this person-time from the analysis, including potential cases of lymphoma that should have been attributed to the unexposed group, thus amplifying artificially the risk for all treatment groups. Even assuming that such a loss of person-time did not delete any case of lymphoma, the exposure wrongly attributed to the treatments in the main analysis was likely to be depleted, at least in part. Indeed, if the exposure time in the denominator of an exposed group is reduced, the consequence will be an artificial magnification of the frequency of adverse events (i.e., lymphoma). Therefore, the ultimate effect of such a person-time loss in the exposed groups is likely a confirmation (or even an amplification) of the risk estimated in the main analysis. The second study supporting an association was that by Herrinton et al. (2011), which apparently made a similar mistake in the definition of the exposure. The correct way to define the exposure would be from the cohort entry (first intake of the drug) up to the censoring point (outcome of interest, death, end of the study period, loss to follow-up). The person-time included in this period should be attributed to the exposed group. Herrinton et al. attributed the person-time elapsed after the discontinuation of the anti-TNF treatment to the unexposed group. Consequently, the person-time attributed to the exposed group (denominator) strongly concentrated, thus increasing the frequency of lymphoma and resulting in an apparent increase of the risk. In support of this hypothesis, it is easy to verify that the person-time attributed to the exposed group in this study was the 0.9% of the overall person-time of the cohort.

The choice of investigating the risk of lymphoma within a broad composite endpoint including different cancers is biologically questionable. Indeed, the pathophysiological mechanisms supporting the development of cancer are extremely variable across the different types of cancers and it is therefore unlikely that a single drug can trigger all these mechanisms. Furthermore, several studies postulated, even though without any supportive evidence, that lymphoma could be a class effect of anti-TNF therapy. Such a clustering of endpoints and exposures seems to be often a choice driven by the need of increasing the power of the sample (especially in monocentric studies performed on small databases), disregarding any scientific rationale. Of note, in the two studies (Biancone et al., 2016; D'Haens et al., 2017) supporting an association of anti-TNF drugs with a group of cancers including lymphoma, the net contribution of lymphoma to the overall risk was not assessable and likely negligible [7% (Biancone et al., 2016) and 18% (D'Haens et al., 2017) of the all number of cancers]. Furthermore, the results of both studies are poorly reliable since they included prevalent patients and did not consider a lag period.

Notably, the association of anti-TNF drugs with lymphoma should be considered in light of the biological plausibility. TNF is a cytokine involved in systemic inflammation and modulation of immune system, and its role in the inhibition of carcinogenesis is well-known. Therefore, the inhibition of TNF would be expected to favor neoplastic processes (Aggarwal et al., 2012). Despite this, the risk of lymphoma associated with anti-TNF treatments has not been yet conclusively demonstrated in RA, mainly due to the intrinsic risk of lymphoma associated with this disease (Dias and Isenberg, 2011; Baecklund et al., 2014; Mercer et al., 2017). Since the evidence supporting a risk of lymphoma in IBDs is scarce (Baecklund et al., 2014), in these patients, it should be easier to demonstrate an association, if any, between anti-TNF drugs and lymphoma. However, even in IBD patients, such a risk remains undetermined. Based on this consideration, one might speculate that the association of anti-TNF treatment with lymphoma is unlikely. Nevertheless, differences in the pathophysiological patterns of RA and IBDs might likely play a role in the development of lymphoma and therefore we cannot exclude that this could be the case even under anti-TNF inhibition. On the other hands, we cannot exclude also that TNF inhibition might promote lymphoma in RA but not in IBD, since differences among their background inflammatory conditions could play a role in determining a differential risk of lymphoma.

The present systematic review has some limitations. First, we did not include unpublished studies that could have provided good evidence of an association between anti-TNF drugs and lymphoma. Second, the assessment of methodological limitations was not based on validated tools, but only on the judgment of experts. A standardized evaluation of the quality of the studies could have provided interesting information. However, we do not believe that the above limitations may affect significantly the conclusions of the present review.

## Conclusions

At present, the available observational studies, considering those supporting an association and those not, are biased by methodologic shortcomings and their results are not reliable. Thus, current evidence from observational studies does not allow excluding or confirming an association of lymphoma with the exposure to anti-TNF treatments in IBD patients. Additional well-designed observational studies are warranted to provide a conclusive answer to this relevant question. Moreover, it would be important also to stimulate meta-research studies, intended as critical appraisals of available evidence, particularly that coming from observational studies, to avoid overemphasis on biased results.

## Author Contributions

SF, LL, and IC reviewed the articles and wrote the manuscript. CB and MT reviewed the manuscript and served as supervisors of the reviewing process.

### Conflict of Interest Statement

The authors declare that the research was conducted in the absence of any commercial or financial relationships that could be construed as a potential conflict of interest. The handling editor declared a shared affiliation, though no other collaboration with the author MT at time of review.
